# Constructing and Validating High-Performance MIEC-SVM Models in Virtual Screening for Kinases: A Better Way for Actives Discovery

**DOI:** 10.1038/srep24817

**Published:** 2016-04-22

**Authors:** Huiyong Sun, Peichen Pan, Sheng Tian, Lei Xu, Xiaotian Kong, Youyong Li, Dan Li, Tingjun Hou

**Affiliations:** 1 College of Pharmaceutical Sciences, Zhejiang University, Hangzhou, Zhejiang 310058, P. R. China; 2 State Key Lab of CAD&CG, Zhejiang University, Hangzhou, Zhejiang 310058, P. R. China; 3 Institute of Functional Nano and Soft Materials (FUNSOM), Soochow University, Suzhou, Jiangsu 215123, P. R. China

## Abstract

The MIEC-SVM approach, which combines molecular interaction energy components (MIEC) derived from free energy decomposition and support vector machine (SVM), has been found effective in capturing the energetic patterns of protein-peptide recognition. However, the performance of this approach in identifying small molecule inhibitors of drug targets has not been well assessed and validated by experiments. Thereafter, by combining different model construction protocols, the issues related to developing best MIEC-SVM models were firstly discussed upon three kinase targets (ABL, ALK, and BRAF). As for the investigated targets, the optimized MIEC-SVM models performed much better than the models based on the default SVM parameters and Autodock for the tested datasets. Then, the proposed strategy was utilized to screen the Specs database for discovering potential inhibitors of the ALK kinase. The experimental results showed that the optimized MIEC-SVM model, which identified 7 actives with IC_50_ < 10 μM from 50 purchased compounds (namely hit rate of 14%, and 4 in nM level) and performed much better than Autodock (3 actives with IC_50_ < 10 μM from 50 purchased compounds, namely hit rate of 6%, and 2 in nM level), suggesting that the proposed strategy is a powerful tool in structure-based virtual screening.

Virtual screening (VS) exhibits undefeatable advantage in today’s drug discovery campaign^1^,^2^,^3^, which shows short development time, low financial cost, whereas high production ratio^4^,^5^. Roughly, the VS approaches can be divided into two categories: ligand-based and structure-based strategies^6^. The ligand-based VS approaches employ ligand properties, such as molecular weight, number of hydrogen bond donors/acceptors, solvent accessible surface area, various molecular fingerprinting, etc., to construct prediction models according to known actives. Whereas the structure-based VS approaches additionally employ the target information for the predictions of actives, such as molecular docking, which can give the binding information of ligands upon their targets, *i.e*. the binding poses, the binding affinities, and even the residue-ligand interaction details. In principle, the compounds derived from the ligand-based VS approaches may usually be limited in the scope of similar core fragment molecules due to the reason that the ligand-based VS approaches can only depend on known actives. Whereas, the structure-based VS approaches may find complete new lead compounds by considering the target information. Thereafter, the structure-based VS approaches are more feasible in finding new lead compounds compared with the ligand-based VS approaches^7^,^8^,^9^,^10^.

Up to date, numerous strategies based on ligand-based and structure-based approaches have been proposed for VS. For instance, Sato *et al.* put forward a ligand-based VS strategy by combining three-dimensional molecular shape overlap method and support vector machine (SVM) to evaluate 15 drug targets and gained much better results compared with other two-dimensional structure-similarity based VS strategies^11^. Kong *et al.* developed a biologically relevant spectrum by considering the structures of the primary metabolites of organisms^12^, and found it effective in classifying launched drug from other phase candidates^13^. Our group has proposed a structure-based VS strategy by combining multiple protein structures, including crystallized structures and structures generated by molecular dynamics (MD) simulations, and machine leaning approaches^6^,^14^. Besides, we have also developed a unique structure-based VS approach by combining residue-ligand interaction matrix (also known as Molecular Interaction Energy Components, MIEC) and SVM to discriminate the binding peptides from the non-binders for protein modular domains^15^, and the prediction results have been validated by various experiments^16^,^17^. Since the residue-ligand interaction network can totally reflect the binding specificity of a ligand to the target, we can construct the classification models based on machine learning approaches to discriminate small molecular actives from non-actives. Fortunately, some pioneering work have engaged in this subject, for example, Ding *et al.* have evaluated the performance of MIEC-SVM in discriminating strong inhibitors of HIV-1 protease from a large database (ZINC database)^18^ and they have successfully predicted the binding of a series of HIV-1 protease mutants to drugs^19^. Nevertheless, the performance of MIEC-SVM needs to be assessed by the predictions to more drug targets and validated by real experiments. Moreover, this approach is parameter-dependent, and therefore the strategy to generate the best MIEC-SVM model needs to be addressed. Here, in conjunction with molecular docking, ensemble minimization, MM/GBSA free energy decomposition, and parameters tuning of SVM kernel function, we discussed how to construct a highly performed MIEC-SVM model in three kinase targets (Fig. 1). The best performed MIEC-SVM model for the ALK system was then used for VS, and the experimental results showed that the optimized MIEC-SVM model had markedly improved screening performance compared with the traditional molecular docking method.

## Materials and Methods

### Dataset Preparation and Processing

To summarize the best strategy for the MIEC-SVM construction, three tyrosine kinase targets were at first used for the evaluation, namely ABL (Abelson tyrosine kinase), ALK (Anaplastic lymphoma kinase), and BRAF (v-Raf murine sarcoma viral oncogene homolog B). The crystal structures of 2HYY (for ABL)^20^, 3LCS (for ALK)^21^, and 3IDP (for BRAF)^22^, were employed for the evaluation due to the good performance of Autodock in reproducing the binding modes of their co-crystallized ligands as shown in Table S1 in Supporting Information. All the inhibitors with IC50 (*K*_i_) < 10 μM were obtained from the BindingDB database^23^. In total, 286, 342, and 402 inhibitors were collected for ABL, ALK, and BRAF, respectively. Although DUD dataset^24^ and other strategies^25^ have been proposed for the decoys construction, we did not try to use these strategies to avoid constructing models with limited range of the chemical properties. Thereby, 7000 compounds randomly chosen from the ChemBridge database by using the *Find Diverse Molecules* protocol in Discovery Studio 2.5 were used as non-inhibitors (or background molecules). The structural diversity was shown in Figure S1, where the structural similarity was calculated between the training dataset and the test dataset for the known inhibitors (Figure S1 A–C) and non-inhibitors (Figure S1G), and also between the known inhibitors and non-inhibitors for each target (Figure S1D–F). To the end, the ratio between inhibitors and non-inhibitors is approximate 1:24, 1:20, and 1:17 for ABL, ALK, and BRAF, respectively.

### Molecular Docking

Autodock 4.2^26^ with Lamarckian genetic algorithm (LGA)^27^ was employed for the docking mode selection due to its good performance of reproduction capability^28^,^29^,^30^, Before molecular docking, the protein targets were prepared with the *Structure Preparation Tool* module in Sybyl-X1.1, which added hydrogen atoms, repaired side-chains of the imperfect crystallized residues, and optimized the steric hindrance of side-chains. The protonation states of the proteins were determined by using PROPKA (version 3.1)^31^. Autodock4 atomic radii and Gasteiger partial charges^32^ were assigned to the macromolecules and the small molecules in molecular docking. The conformation selection space of a ligand was set to 18.75 × 18.75 × 18.75 Å^3^ (corresponding to 50 × 50 × 50 grids, with each grid 0.375 Å in length) around the binding pocket for each target. Each ligand was docked for 10 times to meet the demand of retrieving the top three docking poses with sufficient selection space.

### Molecular Mechanics Optimization

Prior to molecular mechanics optimization, the ligand-protein systems were constructed with *antechamber* and *tleap* modules^33^ in Amber12 simulation package^34^. AM1-BCC charges^35^ were calculated for the small molecules by using *sqm* module in Amber12 due to its good performance and low computational cost^36^,^37^. The cutoff value was set to 8 Å to handle the short range electrostatic and van der Waals interactions, while the Particle mesh Ewald (PME) algorithm was employed to deal with the long-range electrostatic interactions^38^. Amber03 force filed^39^ and General Amber force field (GAFF)^40^ were used for the proteins and small molecules, respectively. Counter-ions of Na^ + ^and Cl^−^ were added to neutralize the unbalanced charges of the systems. Octahedral-shaped TIP3P water box^41^ was added for each ligand-protein complex with 5 Å extended out of the solute to save the computational resources. Three phases of minimization were used to optimize each ligand-protein system. In the beginning, 50 kcal/mol · Å^2^ elastic constant was used to constrain the backbone atoms of protein for 1000 cycles (500 cycles of steepest descent and 500 cycles of conjugate gradient minimization); then, the elastic constant was decreased to 10 kcal/mol · Å^2^ for 1000 cycles; finally, the whole system was relaxed without any constrain for 3000 cycles. The optimized structure was submitted for free energy decomposition to derive the energy components.

### Molecular Interaction Energy Components (MIEC) Matrix Calculation

The classifiers based on MIEC have been found effective in discriminating the known binding peptides from non-binders for protein modular domains in the previous studies^16^,^18^,^42^,^43^, Here, the MM/GBSA free energy decomposition approach was employed for the MIEC matrix construction based on the optimized structures. The residue-ligand interactions can be expanded as following:


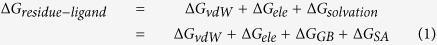


where Δ*G*_residue-ligand_ denotes the total interaction energy between a residue and a ligand, which is composed of four terms: the van der Waal interaction (Δ*G*_vdW_), the electrostatic interaction (Δ*G*_ele_), the polar part of solvation energy (Δ*G*_GB_), and the non-polar part of solvation energy (Δ*G*_SA_). The modified GB model developed by Onufriev *et al.* was employed for the polar solvation energy calculation^44^, and the ICOSA algorithm was employed to estimate the non-polar part of solvation energy^45^. Due to the good performance of a relatively higher interior dielectric constant in kinase systems^46^,^47^, all the free energy decompositions were performed under the interior dielectric constant of 4 (*ε*_in_ = 4). Here, the MIEC matrices were constructed by using two strategies based on the top 1 docking pose and the best of the top three docking poses due to the fact that the rescoring process may re-rank the originally scored binding modes of the small molecules^48^,^49^. For the later strategy, MM/GBSA was at first used for rescoring of the top three docking poses (*ε*_in_ = 4)^47^, and then, the best rescored binding pose (of the three docking poses) of each system was employed for MM/GBSA decomposition.

### MIEC-SVM Model Construction

The energy components were selected and combined (such as the combinations of Δ*G*_ele_ and Δ*G*_vdW_; Δ*G*_GB_ and Δ*G*_SA_; Δ*G*_ele_ and Δ*G*_GB_; Δ*G*_vdW_ and Δ*G*_SA_; and Δ*G*_ele_, Δ*G*_vdW_, Δ*G*_GB_, and Δ*G*_SA_, as shown in Tables S2–S4) to construct the MIEC matrices, where the energy components of the vital residues for the inhibitor binding, *i.e.* the top 20, 25, and 30 contributed residues (that is, 40, 50, and 60 vectors for the combinations of Δ*G*_ele_ and Δ*G*_vdW_, Δ*G*_GB_ and Δ*G*_SA_, Δ*G*_ele_ and Δ*G*_GB_, Δ*G*_vdW_ and Δ*G*_SA_, and 80, 100, and 120 vectors for the combination of Δ*G*_ele_, Δ*G*_vdW_, Δ*G*_GB_, and Δ*G*_SA_; and the top residues were chosen by adding all the residue-ligand interaction pairs for all the known inhibitors, averaging the total energies for each residue-ligand pair according to the number of known inhibitors, and sorting the averaged residue-ligand pair for each target), were selected as the eigenvectors for the classification of inhibitors and non-inhibitors. Herein, the SVM algorithm^50^,^51^ implemented in *libsvm* package^52^ was employed for the model construction. Although SVM has been widely used in the scope of drug design due to the high accuracy of nonlinear fitting^53^,^54^,^55^, it may miss the best model if without the hyper-parameters optimization, such as when using the Radial Basis Function (RBF) as the kernel function, the parameters *cost* (*c*, which scales the penalty of classifying the samples to a false group) and *gamma* (*γ*, which regulates the inverse radius of influence of the training samples) should be adjusted carefully because they may significantly affect the classification performance^56^. Herein, RBF was also used as the kernel function, and grid searching was employed for the parameter tuning for the purpose of finding the best Matthews correlation coefficient (*MCC*) of the training dataset. The *c* and *γ* values were designed exponentially growing against 2, namely 2^*n*^, where *n* denotes *c* or *γ* and goes from −2 to 10 and −10 to 2, respectively. The grid space was set to 0.5 for both *c* and *γ*. Thereby, a total of 525 models were constructed for each protocol (Fig. 2, which will be discussed below). In searching different values of *c* and *γ*, different MCC values will be produced (as shown in Fig. 2), and the *c* and *γ* values corresponding to the highest MCC value were termed as best *c* and best *γ*, respectively, which were used for the external test dataset validation (Tables S2–S4). Here, half of the molecules (half inhibitors and non-inhibitors for each target) were randomly selected as the training set for model construction and the remaining molecules were used as the external test set for model verification. To assess the statistical significance of the classifiers, 5-fold cross-validation was employed for the validation of each model (or grid). Due to the unbalance of the inhibitors and non-inhibitors (~1:20), a higher weight (1.2) was set for the inhibitors to balance the classification^42^.

**Figure 1:**
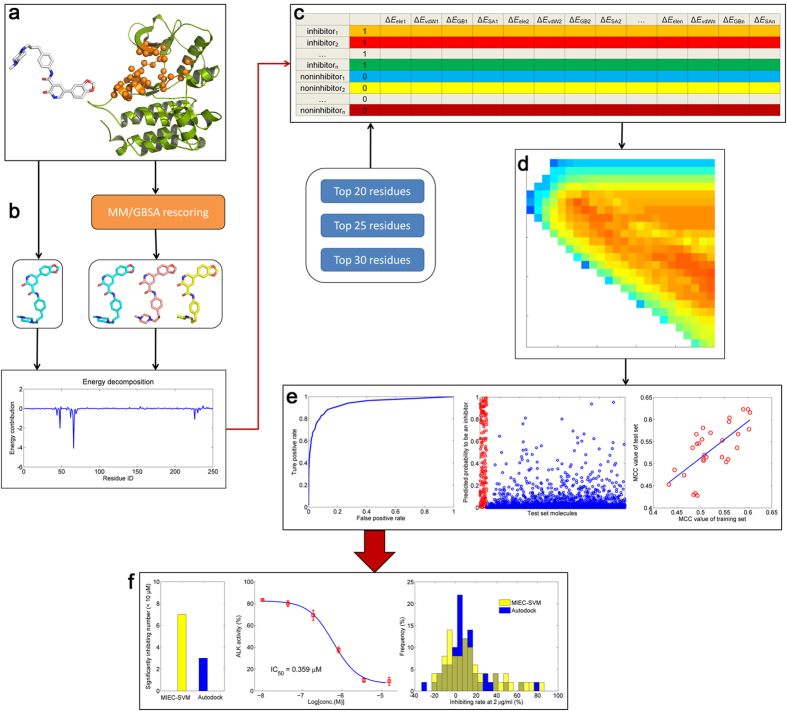
Workflow of the MIEC-SVM based classification model construction and experimental testing. (**a**) molecular docking, the most contributed residues were colored in orange; (**b**) residue decomposition, two strategies were used here: the top 1 docking pose was directly used for energy decomposition; and the top three docking poses were at first rescored by MM/GBSA approach, and then the best rescored docking pose was used for the decomposition analysis; (**c**) MIEC matrix construction, different combinations of energy components and top contributed residues were used for the matrix construction; (**d**) hyper-parameters optimization, *c* and *γ* were tuned using the grid searching approach and the corresponding MCC values were colored from blue (bad performance) to red (good performance); (**e**) model evaluation, the ROC curve, inhibitor probability, and Pearson correlation coefficient were employed for the model evaluation; (**f**) experimental testing, compound activity enrichment, enzyme inhibitory rate distribution, and the IC_50_ curves were used for the comparison of the methodologies.

To evaluate the performance of the MIEC-SVM models, besides the MCC values, the sensitivity (*SE*), specificity (*SP*), prediction accuracies for inhibitors (*Q*_+_) and non-inhibitors (*Q*_−_) shown in Equations (2)~(6) were also considered for the comparison.


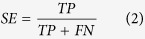



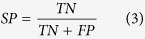



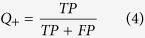



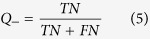



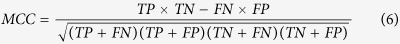


where *TP* and *FP* denote the number of true positives (inhibitors) and false positives, and *TN* and *FN* represent the number of true negatives (non-inhibitors) and false negatives. Moreover, the *AUC* value (area under curve) of the ROC (receiver operating characteristic) curve was also employed to quantitatively evaluate the prediction accuracy of each model based on the probability of a molecule to be an inhibitor given by the support vectors. The workflow of the data processing, model construction, and experimental testing was briefly summarized in Fig. 1.

### Virtual Screening and Compounds Selection

To evaluate the performance of the optimized MIEC-SVM model in real experiments, the ALK system was used for the experimental testing. Here, the best performed MIEC-SVM model (model 15 in Table S3 with top 1 docking pose) for the ALK system was used to virtually screen the Specs database (containing ~220,000 compounds). Due to the high computational cost of the MIEC matrix calculation, herein, a hierarchical strategy was used for virtual screening: (1) all the compounds in Specs were docked into the binding site of ALK by Autodock 4.2 and scored by the Autodock scoring function; (2) the top 30,000 molecules ranked by the Autodock score were extracted for the MIEC matrix calculation and scored by the optimized MIEC-SVM model; (3) the top 300 compounds ranked by the MIEC-SVM model (inhibitor-probability derived from the SVM algorithm) and Autodock score were then respectively filtered by Lipinski’s “rules of five” (compounds with violation number ≥ 2 were eliminated) and the drug-likeness model developed by our previous study^57^,^58^; (4) in order to maximize the chemical diversity of the collected compounds for bioassays, the remaining compounds were structurally clustered, and the compounds with the Tanimoto similarity matrix computed from the *MACCS* structural keys higher than 0.80 were clustered into the same group^59^. The top molecule in each group was then sorted by the inhibitor-probability based on the MIEC-SVM model or the docking score based on Autodock. Finally, the top 50 compounds in each group (ranked by the MIEC-SVM probability and the Autodock docking score) available from Specs were purchased for experimental testing (purity ≥ 95%, confirmed by Specs, Table S5).

### Reagents and Materials for Bioassays

All reagents and anhydrous solvents were obtained from commercial sources and used as received. The positive control inhibitors of ALK, crizotinib and ceritinib, were purchased from Bangshunda Technology and Selleck Chemicals, respectively. The compounds were dissolved in 100% dimethyl sulfoxide (DMSO) as a 10 mM stock solution. The final DMSO concentration in each reaction was less than 1%. Purified recombinant human ALK protein (Catalog number: PV3867) as well as reagents for TR-FRET assay, including Lantha Screen™ Tb-PY20 (Catalog number: PV3552), Fluorescein-Poly GT (Catalog number: PV3610), ATP (Catalog number: PV3227), Kinase Quench Buffer (Catalog number: P2832), Kinase Buffer (Catalog number: PV3189), and Antibody Dilution Buffer (Catalog number: PV3574), were all obtained from Life Technologies Inc.

### *In vitro* Inhibitory Activity Assay of ALK

Lantha Screen™ kinase assay based on TR-FRET technology was used to measure the inhibitory activity of the screened compounds. All the assays were carried out in 384-well plate format. The 4 × test compounds were firstly prepared before the enzyme reaction starts. The 4 × recombinant human ALK protein and 2 × Substrate/ATP mixture were separately prepared in 50 mM HEPES (pH = 7.5), 0.01% BRIJ-35, 10 mM MgCl_2_, 4 mM MnCl_2_, 1 mM EGTA, and 2 mM DTT. The final 10 μL kinase reaction consists of 5 μL 1 × Substrate/ATP mixture (0.2 μM substrate and 5 μM ATP), 2.5 μL ALK (5 ng/ml ALK protein), and 2.5 μL 1 × test compounds with desired concentration. The assay plate was shook on a plate shaker for 30 seconds to mix the reactions thoroughly. After 1 hour kinase reaction incubation at room temperature (20 ~ 25 °C), 10 μL of pre-prepared 20 mM EDTA and 4 nM Tb-labeled antibody solution was then added to terminate the kinase reactions and to initiate antibody binding, and the assay plate was incubated for another 1 hour at room temperature. Then, the assay plate was placed into a fluorescence plate reader (BioTek Synergy™ 4) to measure both fluoresce in and terbium emission signals (excitation: 340 nm; emission: 520 and 495 nm, respectively) with 100 μs delay time and 200 μs integration time. To determine the IC_50_ values, the resulted inhibitory activity calculated from TR-FRET emission ratio (i.e. fluoresce in emission intensity/terbium emission intensity) was plotted against the concentration of inhibitor, and the data was fitted to a dose-response curve with a variable slop.

## Results and Discussion

### Vital Residues for Model Construction

To construct an effective classification model, distinguishable features should exist between the positive and negative samples, though it may be hard to be discriminated by simple observations. In the spirit of MIEC-SVM model, it considers only the binding specificity of the known inhibitors and non-inhibitors rather than the chemical structures of the known inhibitors. The model can discriminate the specific energetic spectra of the systems with even very little difference, such as a series of similar drug derivatives against a same protein^60^,^61^,^62^, a same drug against homologous proteins (drug selection)^63^,^64^ or protein mutants (drug resistance)^65^,^66^,^67^, etc. Therefore, the MIEC-SVM model is superior to most of the ligand/structure-based methods that usually bias the models to screen some known structures in the training set.

Here, the energetic contributions based on vital ligand-residue pairs were employed as the classification features to discriminate the inhibitors from non-inhibitors. The energy contributions of the top 11 contributed residues (within ~4 Å of the co-crystallized ligands for the three targets) to the inhibitors and non-inhibitors for the three targets were averaged and shown in Figure S2. Apparently, the energy contributions of the vital residues to the inhibitors (red bar) are always larger than those to the non-inhibitors (green bar) for all the three systems, implying that it is intrinsically distinguishable between the inhibitors and non-inhibitors by using the molecular interaction energy components contributed from the vital residues.

### Importance of Tuning Hyper-parameters in MIEC-SVM Model Construction

**Figure 2:**
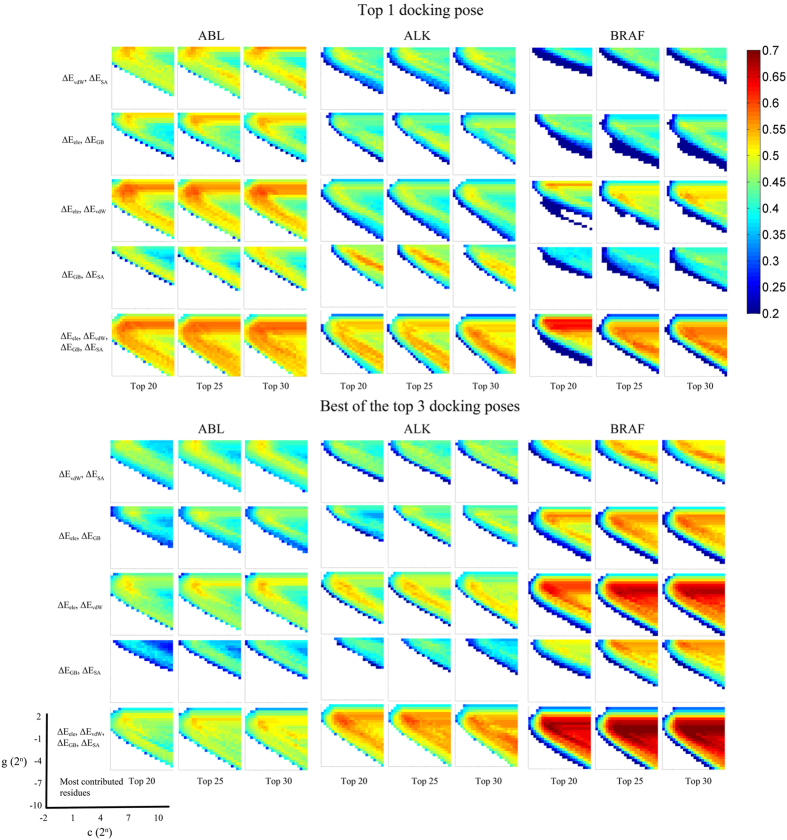
Parameter optimization of the training set. The MCC values are plotted from blue to red. The different top contributed residues, docking poses, and combinations of energy components are employed to give a comparison.

As has been discussed above, two hyper-parameters (*c* and *γ*) may significantly affect the prediction performance of the models when using the RBF kernel function^56^. However, numerous studies ignored this key step by just using the default parameters (such as in *libsvm c* = 0 and *γ* = log_2_(1/n_features), with 2^*n*^ in unit)^18^,^19^,^42^,^43^,^54^, and thereafter may loss the best model. Here by using the grid searching approach, the *c* and *γ* values were optimized by finding the grid with the highest MCC value to construct the best prediction models. Afterward, the best models were validated by the predictions to the prior prepared test sets. As shown in Fig. 2, remarkable difference was observed of the MCC values when using different combinations of the *c* and *γ* values for the training set. The MCC values of the training set vary from 0.2 (blue grids) to 0.7 (red grids) for the different groups of *c* and *γ* (grids were not colored when the predicted MCC less than 0.2). By using different combinations of energy components and docking poses (will be discussed in the following), 30 best performed MIEC-SVM models were constructed for each target as shown in Tables S2–S4. Interestingly, there is no best models located in the grid of *c* = 0 (or 2^*c*^ = 1), implying that it may miss the best choice to construct the SVM classifiers based on the default parameters.

**Figure 3:**
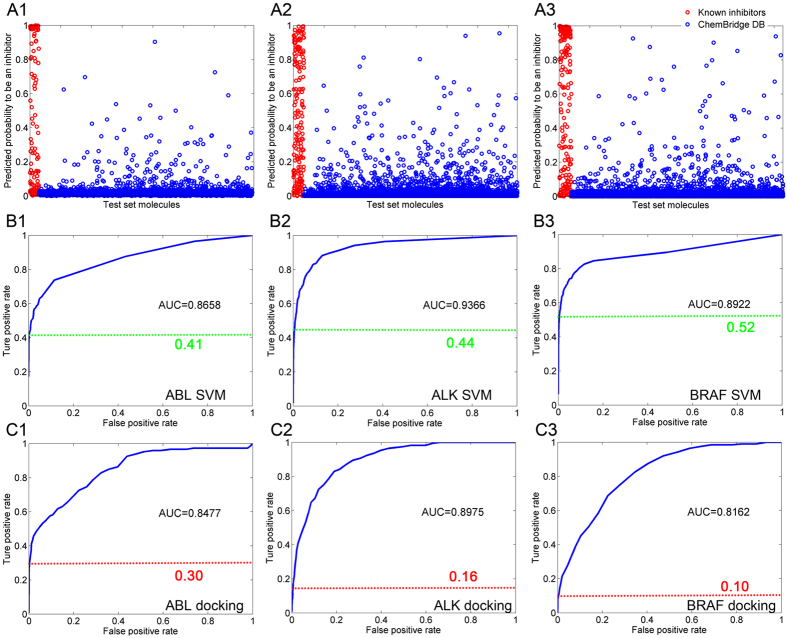
Comparison of the ROC curves of the SVM probabilities and the docking poses for the test set of the three systems. The probability of a molecule to be an inhibitor is plotted in panel (**A**), where the inhibitors and the non-inhibitors are colored in red and blue, respectively. The ROC curves based on SVM probabilities (the inflection points are shown in green dot line) and docking scores (the inflection points are shown in red dot line) are illustrated in panel (**B**,**C**), respectively. The inflection points were measured by 1% false positive rate (Considering the test set contains 3500 non-inhibitors, the point with 35 non-inhibitors classified into the inhibitor group was used to determine the inflection point position).

The correlation of the top 30 models for each target is plotted in Figure S3. Although the MCC values are large for the models based on the different combinations of the selected classification features (as illustrated in Tables S2–S4), high correlation coefficients were found across all the systems (*r* = 0.7 ~ 0.9), meaning that the models in each combination are stable and not over-fitted. Thereby, it is reliable to analyze the predicted results of the external test set based on the best model for each target (herein, the best model was chosen based on the highest training set MCC values for each target). The probability of a molecule to be an inhibitor was estimated by the SVM model as shown in Fig. 3A1–A3, where model 15 of ABL (based on the top 1 docking pose strategy), model 5 of ALK (based on the best of the top three docking poses strategy), and model 15 of BRAF (based on the best of the top three docking poses strategy) are plotted. It can be found that most of the non-inhibitors (blue cycles) are located at the bottom of the figures (Fig. 3A1–A3), suggesting that a large part of the molecules are recognized as in-actives, which is consistent with the fact that only few compounds natively show activities to the drug target. The corresponding ROC curve for each test set was also calculated based on the probability as shown in Fig. 3B1–B3, where the inflection points are shown in green dot lines (the inflection points were measured by 1% false positive rate of the test set as the fact that actives always exist in the chemical background, though the ratio is very low, usually < 1%).

To give a comparison, the ROC curve for the test set based on the top 1 docking scores of the inhibitors and non-inhibitors were also plotted for each target (Fig. 3C). As shown in Fig. 3B,C, the AUC values based on the best MIEC-SVM models (0.866 for ABL, 0.937 for ALK, and 0.892 for BRAF, Fig. 3B1–B3) are all significantly higher than the corresponding results based on the top 1 docking scores (0.848 for ABL, 0.898 for ALK, and 0.816 for BRAF, Fig. 3C1–C3), with the AUC values increased by ~2% for ABL, ~4% for ALK, and ~8% for BRAF. Besides, it shows that the inflection points of the ROC curves based on the SVM probabilities (green dot lines in Fig. 3B) are also much higher than the corresponding docking results (red dot lines in Fig. 3C) (0.4 ~ 0.5 *versus* 0.1 ~ 0.3 for the MIEC-SVM models and molecular docking, respectively), meaning that there is more opportunity to find more inhibitors with much lower false positive rate by using the hyper-parameters-tuned MIEC-SVM models. Taken all, considering that a little improvement of the model accuracy (*i.e.* 1%) will remarkably decrease the false positive rate of VS using a large database, it should be a good choice to use the hyper-parameters-tuned MIEC-SVM models for structure-based VS.

### High Quality Model Construction by using More Energy Components

As the fact that the performance of a prediction model is usually affected by multiple factors such as the selection of different combination of feature vectors^14^,^42^,^56^,^68^, apart from tuning the kernel function parameters, we also optimized the MIEC-SVM models by considering different combinations of the feature components, such as different number of most contributed residues (top 20, 25, and 30 residues), different docking poses (top 1 docking pose and the best of the top three docking poses), and different energy components (Δ*G*_ele_ and Δ*G*_vdW_; Δ*G*_GB_ and Δ*G*_SA_; Δ*G*_ele_ and Δ*G*_GB_; Δ*G*_vdW_ and Δ*G*_SA_; and Δ*G*_ele_, Δ*G*_vdW_, Δ*G*_GB_, and Δ*G*_SA_). Although Ding *et al.* found that the use of the top 30 most contributed residues may be the best choice to construct the MIEC-SVM models for the HIV-1 protease^18^, we show here that there is no remarkable difference when using the top 20, 25, or 30 most contributed residues to construct the MIEC-SVM models for the tyrosine kinase systems. As shown in Fig. 2, similar distribution patterns of the MCC values were found across all the groups using different numbers of top contributed residues (here, the three panels in each line within a same target, containing the top 20, 25 and 30 most contributed residues, was considered as a group), indicating that the MIEC-SVM models are not too sensitive to how many top contributed residues are used for model construction (at least for the case that more than 20 top contributed residues were used).

Different from the issue of selecting how many most contributed residues for model construction, the MIEC-SVM models are more sensitive to which energy components are used. As shown in Tables S2–S4, the MCC values are quite different for the training sets. The models based on the combinations using all the four energy components, namely Δ*G*_ele_, Δ*G*_vdW_, Δ*G*_GB_, and Δ*G*_SA_, have better performance than those based on the other combinations of energy components. For instance, in the system of BRAF, the MCC values increase from 0.44 for the combination of Δ*G*_GB_ and Δ*G*_SA_ (model 4 in Table S4) to 0.63 for the combination of the four energy components (model 5 in Table S4). Moreover, as shown in Tables S2–S4, the combinations of Δ*G*_ele_ and Δ*G*_vdW_ always yield better models than those of Δ*G*_ele_ and Δ*G*_GB_ across all the constructed models. The reason why the combinations of Δ*G*_ele_ and Δ*G*_GB_ always perform worse than those of Δ*G*_ele_ and Δ*G*_vdW_ may be attributed to the high correlation between the electrostatic interactions (Δ*G*_ele_) and the polar part of solvation energies (Δ*G*_GB_) upon the binding of small molecules. As shown in Figure S4A1–C1, very high negative correlations (*r* = −0.80 ~ −0.95) between the electrostatic interactions and the polar part of solvation energies exist for most contributed residues among all the three systems (the energies were calculated by summing up the energy components of the most contributed residues in Figure S2 for all the inhibitors and non-inhibitors), implying that much information derived from the features is redundant and it is not sufficient to use just the two features (Δ*G*_ele_ and Δ*G*_GB_) to construct models. On the contrary, there is no obvious correlations (*r* = −0.11 ~ 0.16) between the electrostatic (Δ*G*_ele_) and van der Waals (Δ*G*_vdW_) interactions for the three systems as shown in Figure S4A2–C2, thereby supplying sufficient information for model construction. Nevertheless, the more energy components are used, the more information of features can be included, and the higher quality models can be constructed. One may argue that it may be a better way to normalize or reduce dimensions (such as using principle component analysis, PCA) for the model construction. Considering that the energies calculated here are all based on the same method (or calculating framework) with the same unit (kcal/mol), to keep the explicit physical meaning, we would like not to use any scaling or dimension reduction methods for the model construction.

### The Performance of Using More Docking Poses for Models Construction

As the rescoring process (MM/GBSA) may re-rank the docking poses derived from the original docking results^47^,^49^, we rescored the top three docking poses by the MM/GBSA approach (with the top rescored poses used for model construction). It is well known that docking with multiple pocket conformations derived from different crystal structures is usually superior to one using a single pocket conformation^6^,^14^. However, it will be much time consuming to construct MIEC-SVM models in considering multiple crystal structures. Nevertheless, the optimization of the ligand-receptor complex in the MIEC-SVM model construction actually introduced induced-fit effect upon the ligand binding, which may be superior to the models constructed based on rigid receptor docking. Thereby, herein, two strategies were used for model construction: (1) the top 1 docking poses derived from Autodock were directly decomposed into residue-ligand pairs for model construction; and (2) the top three docking poses were rescored by the MM/GBSA approach at first, and then, the top 1 rescored poses were submitted to energy decomposition for model construction. As shown in the lower half of Fig. 2, which denotes the models derived from the best of the top three docking poses, similar patterns of the MCC distributions were found for the models based on the top 1 docking poses (the upper half of Fig. 2), meaning that the parameter optimization will not depend on which docking pose is used, but the MCC values are very different when using different docking poses. For instance, the best models derived from the top 1 docking pose strategy are mostly better than those derived from the best of the top three docking poses strategy in the system of ABL (Table S2), whereas, in the system of BRAF, the best models derived from the multiple docking poses strategy always perform better than those derived from the top 1 docking pose strategy (Table S4). Thereby, it may be system-specific whether to use multiple docking poses for MIEC-SVM model construction.

### Comparison of the Optimized MIEC-SVM Model and the Traditional Molecular Docking Method based on Experimental Testing

**Figure 4:**
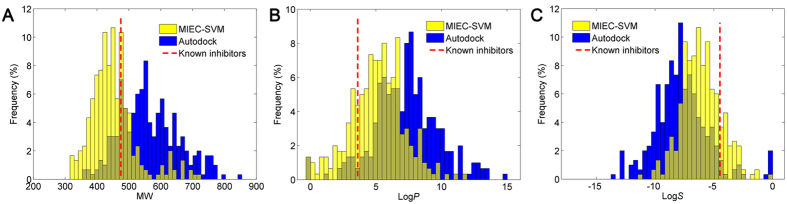
Chemical properties of the top 300 compounds scored by MIEC-SVM (yellow bars) and the top 300 compounds scored by Autodock (blue bars) for ALK. The averaged values of the corresponding chemical properties of the known ALK inhibitors were shown in red dot lines. The distributions of the molecular weight (MW), the predicted octanol/water partition coefficient (log*P*), and the predicted aqueous solubility (log*S*) were shown in panels (**A**–**C**), respectively.

The above issues have discussed how to generate the best MIEC-SVM models for virtual screening. To test whether the optimized MICE-SVM model is really better than the traditional molecular docking methods, the best-performed MIEC-SVM model for the ALK system (model 15 in Table S3) was employed to virtually screen the Specs database. Before experimental testing, we analyzed and compared the distributions of three important molecular properties (molecular weight, octanol/water partition coefficient, and aqueous solubility) for the top 300 molecules predicted by MIEC-SVM and those predicted by Autodock. As shown in Fig. 4, the molecular properties of the top 300 molecules predicted by MIEC-SVM are much closer to the averaged properties of the known ALK inhibitors (red dot lines in Fig. 4). Besides, we analyzed the violation number of Lipinski’s “rules of five” for the top molecules predicted by MIEC-SVM and Autodock. Apparently, as shown in Table 1, most of the top 300 molecules predicted by MIEC-SVM are drug-like compounds (86% with the violation number ≤ 1), while most of the top 300 molecules predicted Autodock are non-drug-like compounds (with only 30.3% exhibiting violation number ≤ 1), suggesting that the molecules predicted by MIEC-SVM are more drug-like than those predicted by Autodock.

**Figure 5:**
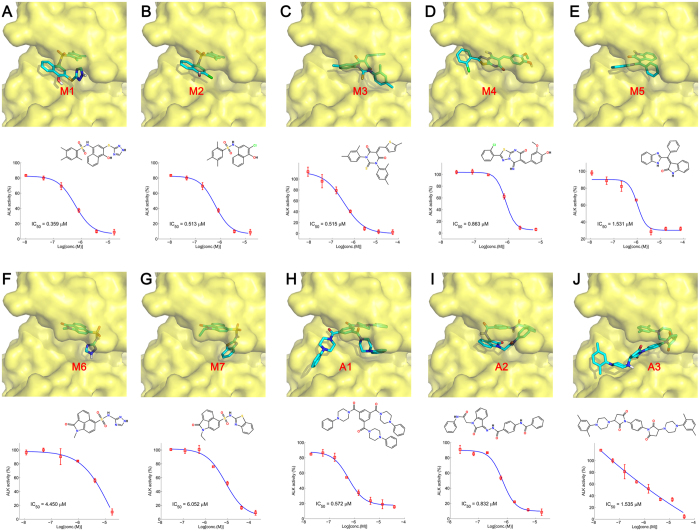
Binding modes and experimental IC_50_ curves of the ALK inhibitors (IC_50_ < 10 μM). The protein and ligands are shown in yellow surface and cyan stick models, respectively. Inhibitors M1-M7 (panels **A**–**G**) were identified by MIEC-SVM model, and inhibitors A1–A3 (panels **H**–**J**) were identified by Autodock 4.2.

Thereafter, the top 50 molecules in each strategy (MIEC-SVM and Autodock methods) remained from the drug-likeness filtering and structural clustering were purchased and submitted to enzyme-based bioassay. Overall, 7 out of 50 (14%) tested compounds chosen by the MIEC-SVM model exhibited remarkable ALK inhibitory activity with IC_50_ < 10 μM (4 molecules showed nM level of activity as shown in Table 2), which is significantly higher than those chosen by Autodock (3 out of 50 molecules, namely hit rate of 6%, and 2 molecules in nM level of activity as shown in Table 2) and consistent with the conclusion that MIEC-SVM model usually goes with higher enrichment ratio (Fig. 3). Moreover, the averaged ALK inhibitory ratio of the tested molecules chosen by MIEC-SVM is also significantly higher than that chosen by Autodock (15% *versus* 9.7%). The binding modes, chemical structures, and IC_50_ curves of the 10 actives (IC_50_ < 10 μM) are shown in Fig. 5.

Besides, to evaluate the novelty of these identified inhibitors with respect to known ALK inhibitors, the pairwise Tanimoto similarity indices based on the FCFP_6 fingerprints for these inhibitors in Table 2 with the known ALK inhibitors obtained from the BindingDB database were calculated through the *Find Similar Molecules by Fingerprints* protocol in Discovery Studio 2.5. It can be found in Table 2 that most of the inhibitors identified by the MIEC-SVM model have low Tanimoto similarity to the known ALK inhibitors (most molecules < 0.2, with only one molecule > 0.3, Table 2), exactly exhibiting the fact that the MIEC-SVM model considers more about the binding specificity of the small molecules rather than the chemical structure of the known inhibitors.

## Conclusion

By using multiple parameters tuning strategy, we systemically evaluated the performance of MIEC-SVM models in discriminating small molecule kinase inhibitors from non-inhibitors. We found that the optimization of the hyper-parameters embedded in the kernel function of SVM is always necessary since the default parameters cannot give the best result for any case of the studied systems. However, it is system-specific whether to use multiple docking poses for MIEC-SVM model construction. Besides, the MIEC-SVM models are not too sensitive to how many energy components are used, such as the models based on the MIEC matrices generated from of the top 20, 25, or 30 residues yield similar prediction capabilities. Whereas, the models are very sensitive to which energy components are employed for model construction, for example, the models based on the combinations of Δ*G*_ele_, Δ*G*_vdW_, Δ*G*_GB_, and Δ*G*_SA_ performed much better than those based on the other reduced combinations. By using the hyper-parameters-tuned MIEC-SVM model, we successfully found 7 significant inhibitors of ALK (IC_50_ < 10 μM) in 50 purchased compounds (with 4 in nM level), suggesting that the hyper-parameters-tuned MIEC-SVM model is a powerful tool for structure-based virtual screening.

## Additional Information

**How to cite this article**: Sun, H. *et al.* Constructing and Validating High-Performance MIEC-SVM Models in Virtual Screening for Kinases: A Better Way for Actives Discovery. *Sci. Rep.* **6**, 24817; doi: 10.1038/srep24817 (2016).

## Supplementary Material

Supplementary Information

## Figures and Tables

**Table 1:** Overall experimental result of MIEC-SVM model and Autodock for ALK system.

	Lipinski violation rate (%)			> 50% inhibitory rate at 2 μg/ml (%)	Averaged inhibitory rate of the purchased 50 molecules (%)	Significantly inhibitory number (<10 μM)
	0	1	≥2			
MIEC-SVM	46.7	39.3	14.0	14	15.0	7
Autodock	3.3	27.0	69.7	6	9.7	3

**Table 2:** Experimentally determined half-maximal inhibitory concentrations (IC_50_) and the corresponding chemical properties of the inhibitors of ALK.

Compound	Specs ID	IC_50_ (μM)	Method	Rank^a^	MW^b^	Log*P*^c^	Log*S*^d^	Similarity^e^
Crizotinib	–	3.33 nM	–	–	450.3	4.73	−2.96	–
Ceritinib	–	3.94 nM	–	–	558.1	3.90	−2.54	–
M1	AQ-390/42708910	0.359	MIEC-SVM	20	440.6	4.09	−5.51	0.140
M2	AQ-390/42425809	0.513	MIEC-SVM	10	375.9	5.44	−6.00	0.165
M3	AN-465/14952108	0.515	MIEC-SVM	19	460.6	6.89	−7.19	0.149
M4	AM-900/40673285	0.863	MIEC-SVM	14	412.9	2.42	−4.47	0.179
M5	AO-080/13867269	1.531	MIEC-SVM	36	337.4	4.30	−5.44	0.367
M6	AQ-390/43364010	4.450	MIEC-SVM	7	329.3	−0.28	−3.15	0.147
M7	AQ-390/40910467	6.052	MIEC-SVM	23	409.5	3.39	−5.27	0.170
M8	AS-871/43476359	11.529	MIEC-SVM	39	435.6	3.52	−5.08	0.183
M9	AO-081/15045283	18.232	MIEC-SVM	41	403.5	3.75	−5.15	0.208
A1	AK-968/15362399	0.572	Autodock	46	642.8	1.30	−3.07	0.173
A2	AG-690/11426045	0.832	Autodock	78	517.6	4.17	−6.17	0.190
A3	AN-919/13953019	1.535	Autodock	118	648.8	3.29	−4.81	0.156

^a^Ranks derived from inhibitor-probability based on MIEC-SVM and docking score based on Autodock.

^b^Molecular weight.

^c^Predicted octanol/water partition coefficient.

^d^Predicted aqueous solubility (*S* in mol/L).

^e^Pairwise Tanimoto similarity indices based on the FCFP_6 fingerprints between each inhibitor and the known ALK inhibitors.
