# Supplementary material for: Constructing and Validating High-Performance MIEC-SVM Models in Virtual Screening for Kinases: A Better Way for Actives Discovery
Source: Sci Rep. 2016 Apr 22;6:24817. doi: 10.1038/srep24817 (PMC4840416; doi:10.1038/srep24817)
Supplement: Supplementary Information [file srep24817-s1.doc]

**Constructing and Validating High-Performance MIEC-SVM Models in Virtual Screening for Kinases: A Better Way for Actives Discovery**

Huiyong Sun,*^a,b,#^* Peichen Pan,*^a,#^* Sheng Tian,*^c,#^* Lei Xu,*^a^* Xiaotian Kong,*^c^* Youyong Li,*^c^* Dan Li,*^a^* and Tingjun Hou*^a,b^,**

^a^College of Pharmaceutical Sciences, Zhejiang University, Hangzhou, Zhejiang 310058, P. R. China.

^b^State Key Lab of CAD&CG, Zhejiang University, Hangzhou, Zhejiang 310058, P. R. China

^c^Institute of Functional Nano and Soft Materials (FUNSOM), Soochow University, Suzhou, Jiangsu 215123, P. R. China.

**Supporting Information**

**
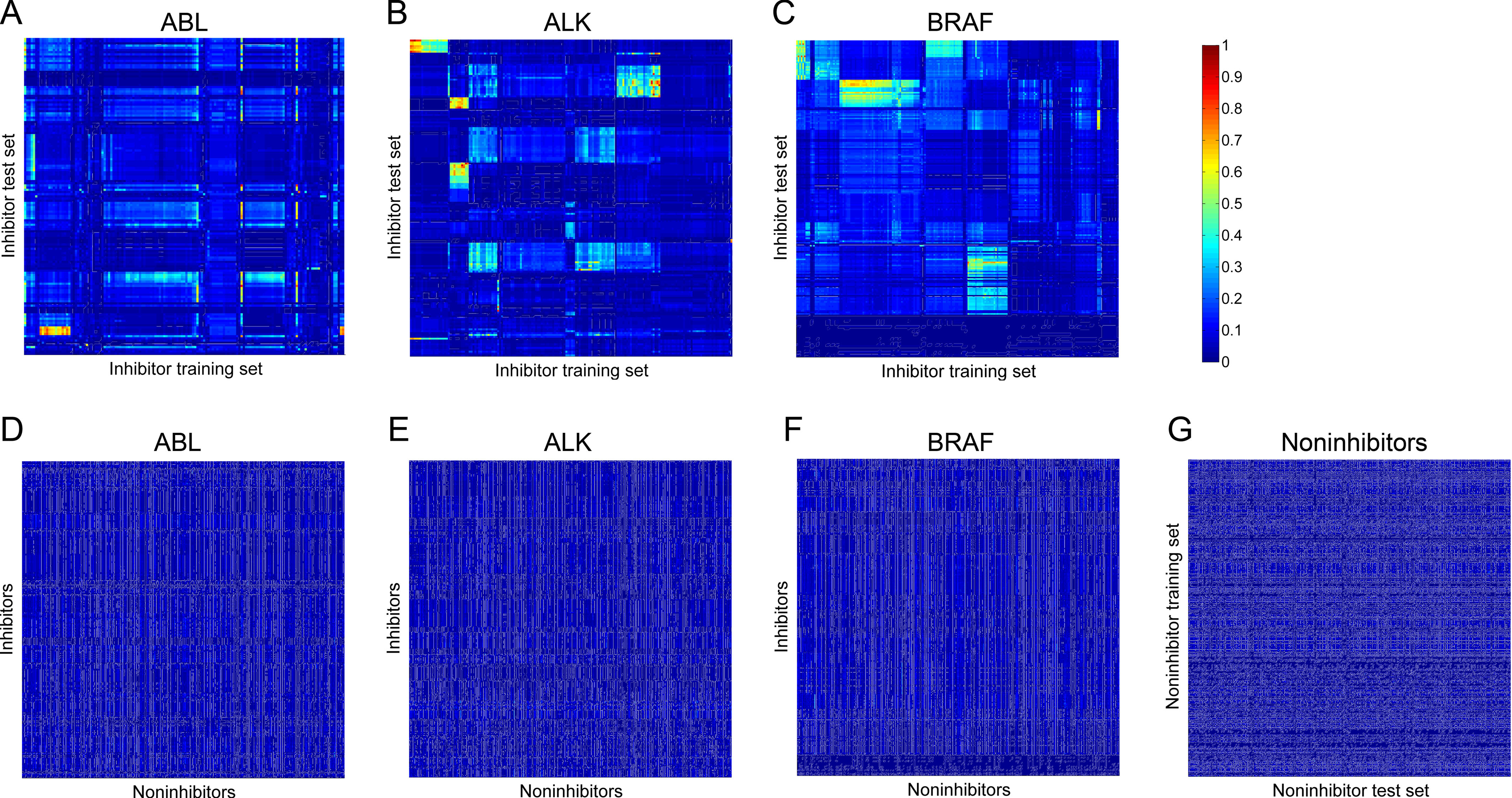
**

**Figure S1.** Structural similarity of the training dataset and the test dataset for the known inhibitors (panels A, B, and C for ABL, ALK, and BRAF, respectively) and the non-inhibitors (panel G). The structural similarity of the known inhibitors and non-inhibitors for each target is also shown in panels D, E, and F for ABL, ALK, and BRAF, respectively.


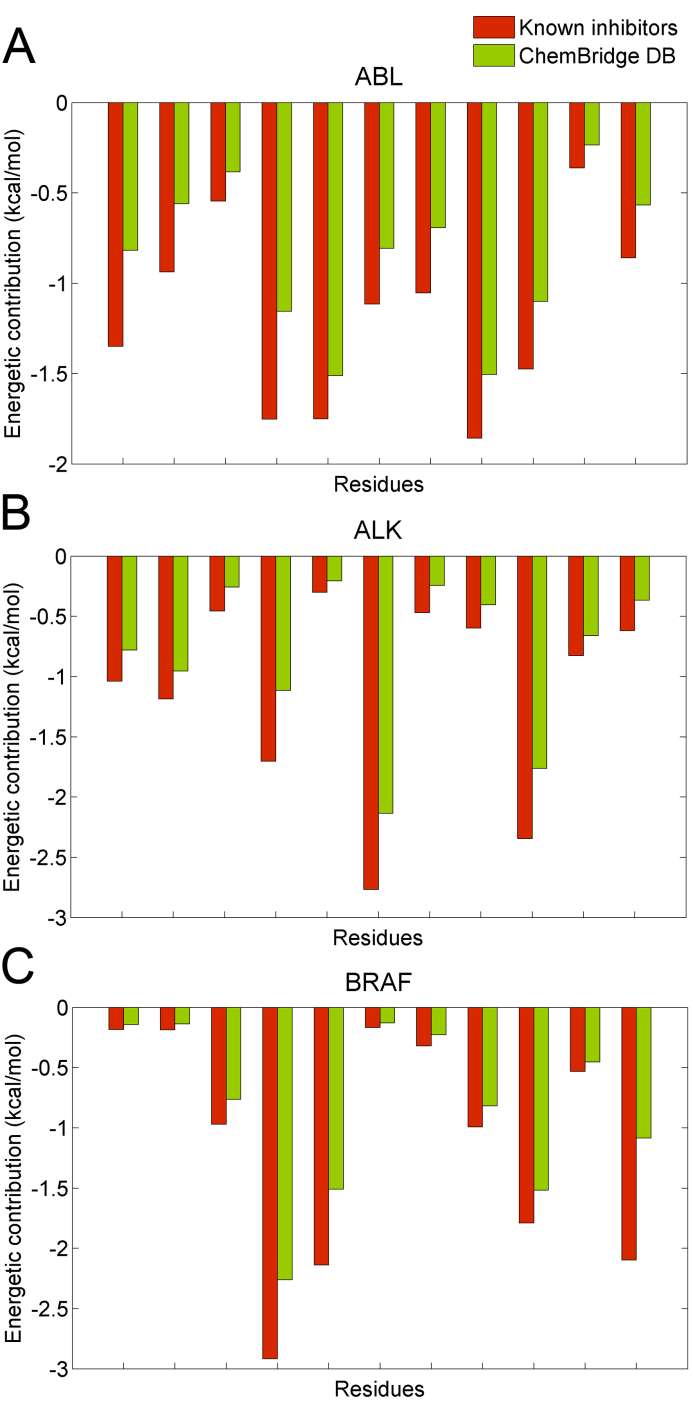


**Figure S2.** Averaged energetic contributions of the most contributed 11 residues between the inhibitors (red) and the non-inhibitors (green) for ABL (A), ALK (B), and BRAF (C).

**
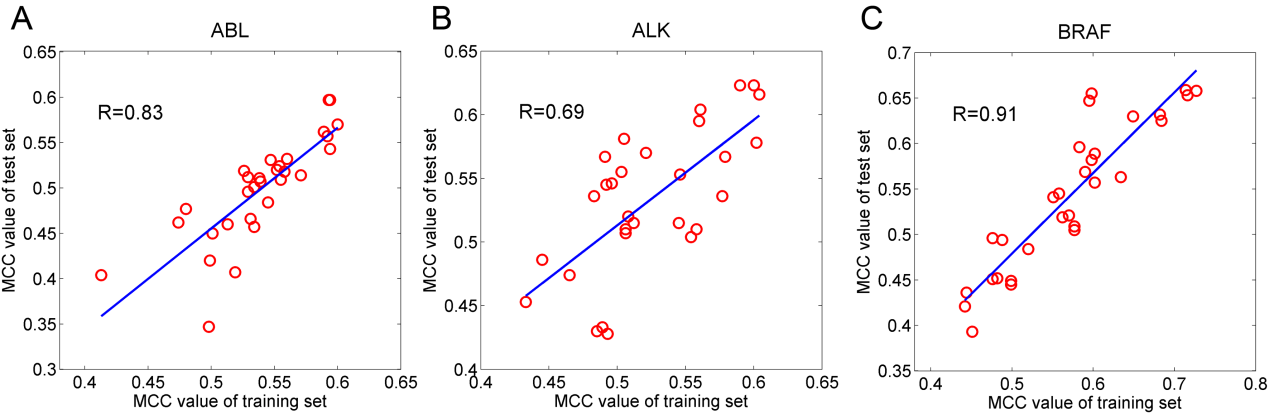
**

**Figure S3.** Stability of the 30 best-preformed models in each term combination of the three systems.

**
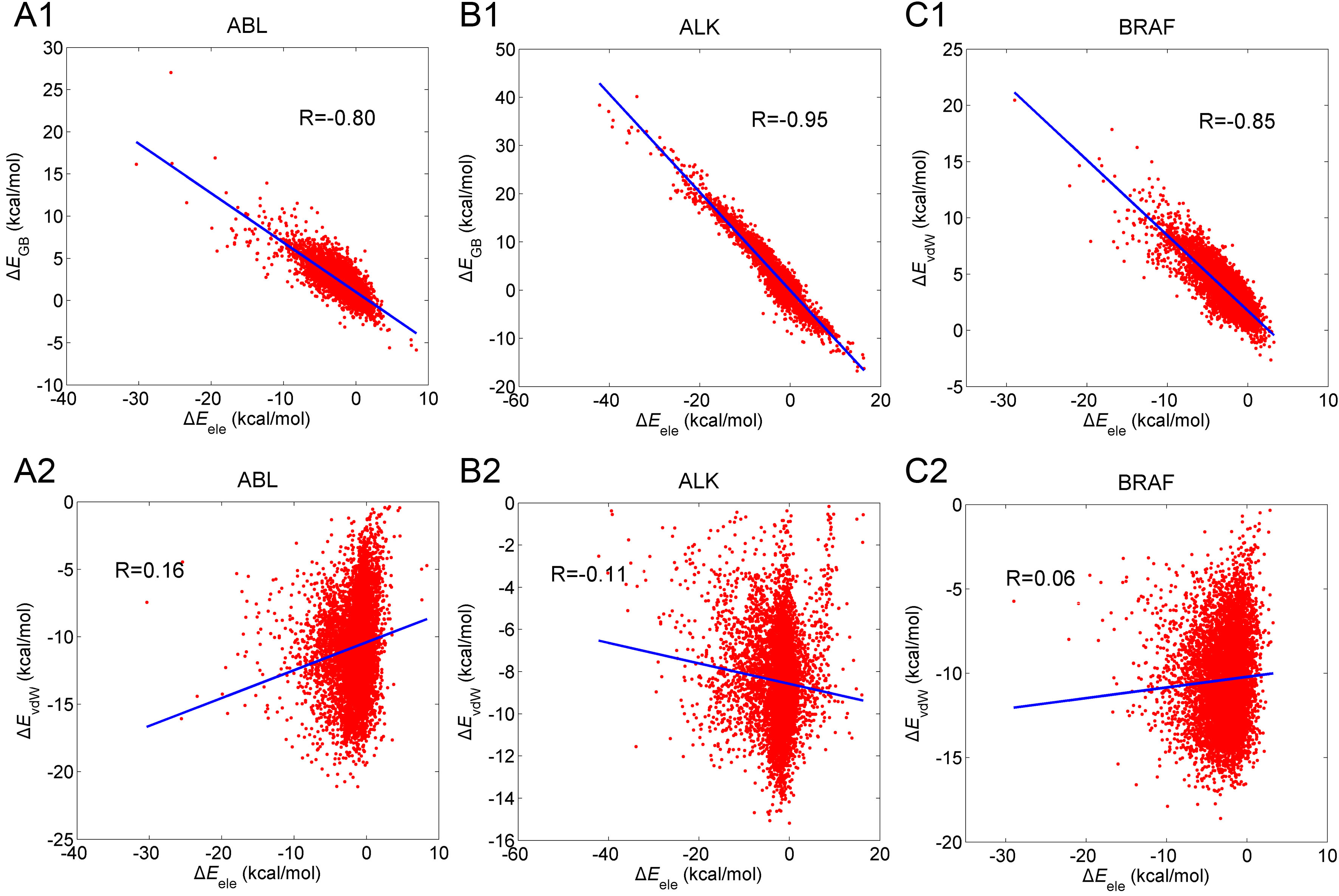
**

**Figure S4.** Correlation coefficients of the total electrostatic (Δ*E*_ele_) and polar part of solvation (Δ*E*_GB_) energies (panels A1-C1), and the total electrostatic and van der Waals (Δ*E*_vdW_) interactions (panels A2-C2).

**Table S1.** Reproduction of the binding modes of the co-crystallized ligands.

| **ABL** | | | **ALK** | | | **BRAF** | | |
| --- | --- | --- | --- | --- | --- | --- | --- | --- |
| **PDB** | **Resolution** | **RMSD** | **PDB** | **Resolution** | **RMSD** | **PDB** | **Resolution** | **RMSD** |
| 2E2B | 2.20 | 0.91 | 2XB7 | 2.50 | 1.16 | 3C4C | 2.57 | 0.59 |
| 2G2H | 2.00 | 1.53 | 2XBA | 1.95 | 1.71 | 3IDP | 2.70 | 0.54 |
| 2HIW | 2.20 | 0.66 | 3LCS | 1.95 | 0.91 | 3PPK | 3.00 | 1.27 |
| 2HYY | 2.40 | 0.25 | 4DCE | 2.03 | 0.89 | 3PRF | 2.90 | 0.54 |
| 2HZI | 1.70 | 0.52 | 4FOB | 1.90 | 1.59 | 4E26 | 2.55 | 4.43 |
| 3CS9 | 2.21 | 0.34 | 4FOC | 1.70 | 2.25 | 4FK3 | 2.65 | 0.72 |
| 3UE4 | 2.42 | 1.66 | 4FOD_1 | 2.00 | 1.45 |  | | |
| 3QRI | 2.10 | 0.59 | 4FOD_2 | 2.00 | 2.46 |
| 3QRK | 2.30 | 0.83 |  | | |

**Table S2.** Performance of various MIEC-SVM models for ABL system.

|  |  | **Top 1 docking pose** | | | | | | | | **Best of the top 3 docking poses** | | | | | | | |
| --- | --- | --- | --- | --- | --- | --- | --- | --- | --- | --- | --- | --- | --- | --- | --- | --- | --- |
| **Model** | **MIECs** | **c (2^c^)** | **γ (2^γ^)** | **MCC_train_** | **SE_test_** | **SP_test_** | **Q_test+_** | **Q_test−_** | **MCC_test_** | **c (2^c^)** | **γ (2^γ^)** | **MCC_train_** | **SE_test_** | **SP_test_** | **Q_test+_** | **Q_test−_** | **MCC_test_** |
| **Top 20 contributed residues** | | | | | | | | | | | | | | | | | |
| **1** | **Δ*G*_vdW_, Δ*G*_SA_** | 0.5 | 1.5 | 0.555 | 0.852 | 0.972 | 0.317 | 0.998 | 0.509 | 1 | 1.5 | 0.513 | 0.844 | 0.970 | 0.262 | 0.998 | 0.460 |
| **2** | **Δ*G*_ele_, Δ*G*_GB_** | 1 | 1 | 0.545 | 0.927 | 0.970 | 0.262 | 0.999 | 0.484 | 2 | 0.5 | 0.498 | 0.692 | 0.967 | 0.186 | 0.997 | 0.347 |
| **3** | **Δ*G*_ele_, Δ*G*_vdW_** | 1.5 | -0.5 | 0.592 | 0.897 | 0.974 | 0.359 | 0.998 | 0.557 | 1.5 | -0.5 | 0.534 | 0.810 | 0.973 | 0.324 | 0.997 | 0.501 |
| **4** | **Δ*G*_GB_, Δ*G*_SA_** | 9 | -4.5 | 0.519 | 0.767 | 0.969 | 0.228 | 0.997 | 0.407 | 2 | 2 | 0.413 | 0.739 | 0.969 | 0.235 | 0.997 | 0.404 |
| **5** | **Δ*G*_ele_, Δ*G*_vdW_, Δ*G*_GB_, Δ*G*_SA_** | 0.5 | -0.5 | 0.594 | 0.958 | 0.972 | 0.317 | 0.999 | 0.543 | 1 | -0.5 | 0.526 | 0.900 | 0.972 | 0.310 | 0.999 | 0.519 |
| **Top 25 contributed residues** | | | | | | | | | | | | | | | | | |
| **6** | **Δ*G*_vdW_, Δ*G*_SA_** | 0.5 | 1.5 | 0.560 | 0.904 | 0.973 | 0.324 | 0.999 | 0.532 | 1 | 1.5 | 0.534 | 0.878 | 0.970 | 0.248 | 0.999 | 0.457 |
| **7** | **Δ*G*_ele_, Δ*G*_GB_** | 1 | 0 | 0.558 | 0.917 | 0.972 | 0.303 | 0.999 | 0.518 | 2 | 0 | 0.501 | 0.809 | 0.970 | 0.262 | 0.997 | 0.450 |
| **8** | **Δ*G*_ele_, Δ*G*_vdW_** | 1 | -1 | 0.594 | 0.965 | 0.975 | 0.379 | 0.999 | 0.597 | 1.5 | -0.5 | 0.539 | 0.880 | 0.972 | 0.303 | 0.998 | 0.507 |
| **9** | **Δ*G*_GB_, Δ*G*_SA_** | 0.5 | 1 | 0.529 | 0.976 | 0.971 | 0.276 | 1.000 | 0.512 | 1 | 2 | 0.480 | 0.925 | 0.970 | 0.255 | 0.999 | 0.477 |
| **10** | **Δ*G*_ele_, Δ*G*_vdW_, Δ*G*_GB_, Δ*G*_SA_** | 0.5 | -0.5 | 0.589 | 1.000 | 0.973 | 0.324 | 1.000 | 0.562 | 0.5 | -0.5 | 0.538 | 0.976 | 0.971 | 0.278 | 1.000 | 0.511 |
| **Top 30 contributed residues** | | | | | | | | | | | | | | | | | |
| **11** | **Δ*G*_vdW_, Δ*G*_SA_** | 0.5 | 1.5 | 0.571 | 0.882 | 0.972 | 0.310 | 0.998 | 0.514 | 1 | 1 | 0.531 | 0.864 | 0.970 | 0.262 | 0.998 | 0.466 |
| **12** | **Δ*G*_ele_, Δ*G*_GB_** | 1.5 | -0.5 | 0.552 | 0.885 | 0.972 | 0.317 | 0.998 | 0.520 | 2.5 | -0.5 | 0.499 | 0.750 | 0.970 | 0.248 | 0.997 | 0.420 |
| **13** | **Δ*G*_ele_, Δ*G*_vdW_** | 1.5 | -1 | 0.600 | 0.887 | 0.975 | 0.379 | 0.998 | 0.570 | 1 | -0.5 | 0.554 | 0.977 | 0.971 | 0.290 | 1.000 | 0.524 |
| **14** | **Δ*G*_GB_, Δ*G*_SA_** | 3 | -1.5 | 0.529 | 0.846 | 0.972 | 0.303 | 0.998 | 0.496 | 1 | 2 | 0.474 | 0.944 | 0.969 | 0.235 | 0.999 | 0.462 |
| **15** | **Δ*G*_ele_, Δ*G*_vdW_, Δ*G*_GB_, Δ*G*_SA_** | 1 | -1.5 | 0.593 | 0.949 | 0.975 | 0.386 | 0.999 | 0.597 | 1 | -1 | 0.547 | 0.920 | 0.972 | 0.317 | 0.999 | 0.531 |

**Table S3.** Performance of various MIEC-SVM models for ALK system.

|  | **Top 1 docking pose** | | | | | | | | | **Best of the top 3 docking poses** | | | | | | | |
| --- | --- | --- | --- | --- | --- | --- | --- | --- | --- | --- | --- | --- | --- | --- | --- | --- | --- |
| **Model** | **MIECs** | **c (2^c^)** | **γ (2^γ^)** | **MCC_train_** | **SE_test_** | **SP_test_** | **Q_test+_** | **Q_test−_** | **MCC_test_** | **c (2^c^)** | **γ (2^γ^)** | **MCC_train_** | **SE_test_** | **SP_test_** | **Q_test+_** | **Q_test−_** | **MCC_test_** |
| **Top 20 contributed residues** | | | | | | | | | | | | | | | | | |
| **1** | **Δ*G*_vdW_, Δ*G*_SA_** | 2 | 0 | 0.506 | 0.710 | 0.970 | 0.386 | 0.992 | 0.507 | 2.5 | 0 | 0.485 | 0.646 | 0.967 | 0.310 | 0.992 | 0.430 |
| **2** | **Δ*G*_ele_, Δ*G*_GB_** | 8 | -4 | 0.483 | 0.648 | 0.974 | 0.474 | 0.987 | 0.536 | 2 | 0.5 | 0.492 | 0.798 | 0.971 | 0.392 | 0.995 | 0.545 |
| **3** | **Δ*G*_ele_, Δ*G*_vdW_** | 1 | -1 | 0.505 | 0.883 | 0.971 | 0.398 | 0.997 | 0.581 | 2.5 | -1 | 0.558 | 0.717 | 0.970 | 0.386 | 0.993 | 0.510 |
| **4** | **Δ*G*_GB_, Δ*G*_SA_** | 9 | -2.5 | 0.579 | 0.686 | 0.976 | 0.497 | 0.989 | 0.567 | 6 | 0 | 0.445 | 0.565 | 0.974 | 0.456 | 0.983 | 0.486 |
| **5** | **Δ*G*_ele_, Δ*G*_vdW_, Δ*G*_GB_, Δ*G*_SA_** | 5.5 | -6.5 | 0.560 | 0.774 | 0.975 | 0.480 | 0.993 | 0.595 | 2 | -1.5 | 0.604 | 0.835 | 0.975 | 0.474 | 0.995 | 0.616 |
| **Top 25 contributed residues** | | | | | | | | | | | | | | | | | |
| **6** | **Δ*G*_vdW_, Δ*G*_SA_** | 2 | 0 | 0.506 | 0.717 | 0.970 | 0.386 | 0.993 | 0.510 | 4 | -0.5 | 0.489 | 0.613 | 0.968 | 0.333 | 0.990 | 0.433 |
| **7** | **Δ*G*_ele_, Δ*G*_GB_** | 9 | -5 | 0.496 | 0.669 | 0.974 | 0.474 | 0.989 | 0.546 | 7.5 | -2.5 | 0.508 | 0.601 | 0.975 | 0.485 | 0.984 | 0.520 |
| **8** | **Δ*G*_ele_, Δ*G*_vdW_** | 1.5 | -1.5 | 0.503 | 0.802 | 0.971 | 0.404 | 0.995 | 0.555 | 2.5 | -1 | 0.554 | 0.702 | 0.970 | 0.386 | 0.992 | 0.504 |
| **9** | **Δ*G*_GB_, Δ*G*_SA_** | 5.5 | -1 | 0.577 | 0.634 | 0.975 | 0.485 | 0.986 | 0.536 | 6 | 0.5 | 0.465 | 0.523 | 0.974 | 0.474 | 0.979 | 0.474 |
| **10** | **Δ*G*_ele_, Δ*G*_vdW_, Δ*G*_GB_, Δ*G*_SA_** | 9 | -8.5 | 0.561 | 0.778 | 0.975 | 0.491 | 0.993 | 0.604 | 2.5 | -1.5 | 0.600 | 0.824 | 0.975 | 0.491 | 0.995 | 0.623 |
| **Top 30 contributed residues** | | | | | | | | | | | | | | | | | |
| **11** | **Δ*G*_vdW_, Δ*G*_SA_** | 2 | 0 | 0.506 | 0.710 | 0.970 | 0.386 | 0.992 | 0.507 | 10 | -2.5 | 0.493 | 0.519 | 0.970 | 0.392 | 0.982 | 0.428 |
| **12** | **Δ*G*_ele_, Δ*G*_GB_** | 2.5 | -0.5 | 0.491 | 0.760 | 0.973 | 0.444 | 0.993 | 0.567 | 6 | -2 | 0.512 | 0.578 | 0.975 | 0.497 | 0.982 | 0.515 |
| **13** | **Δ*G*_ele_, Δ*G*_vdW_** | 1.5 | -1.5 | 0.521 | 0.787 | 0.973 | 0.433 | 0.994 | 0.570 | 1 | -1 | 0.545 | 0.808 | 0.969 | 0.345 | 0.996 | 0.515 |
| **14** | **Δ*G*_GB_, Δ*G*_SA_** | 5.5 | -2.5 | 0.546 | 0.787 | 0.972 | 0.409 | 0.995 | 0.553 | 8 | -1.5 | 0.433 | 0.525 | 0.972 | 0.433 | 0.981 | 0.453 |
| **15** | **Δ*G*_ele_, Δ*G*_vdW_, Δ*G*_GB_, Δ*G*_SA_** | 8 | -7 | 0.590 | 0.740 | 0.978 | 0.550 | 0.991 | 0.623 | 10 | -7 | 0.602 | 0.679 | 0.977 | 0.521 | 0.988 | 0.578 |

**Table S4.** Performance of various MIEC-SVM models for BRAF system.

|  |  | **Top 1 docking pose** | | | | | | | | **Best of the top 3 docking poses** | | | | | | | |
| --- | --- | --- | --- | --- | --- | --- | --- | --- | --- | --- | --- | --- | --- | --- | --- | --- | --- |
| **Model** | **MIECs** | **c (2^c^)** | **γ (2^γ^)** | **MCC_train_** | **SE_test_** | **SP_test_** | **Q_test+_** | **Q_test−_** | **MCC_test_** | **c (2^c^)** | **γ (2^γ^)** | **MCC_train_** | **SE_test_** | **SP_test_** | **Q_test+_** | **Q_test−_** | **MCC_test_** |
| **Top 20 contributed residues** | | | | | | | | | | | | | | | | | |
| **1** | **Δ*G*_vdW_, Δ*G*_SA_** | 6 | 0 | 0.451 | 0.500 | 0.963 | 0.353 | 0.980 | 0.393 | 6 | -1 | 0.558 | 0.634 | 0.972 | 0.508 | 0.983 | 0.545 |
| **2** | **Δ*G*_ele_, Δ*G*_GB_** | 2.5 | 0 | 0.499 | 0.645 | 0.963 | 0.343 | 0.989 | 0.449 | 2 | 0 | 0.583 | 0.869 | 0.968 | 0.428 | 0.996 | 0.596 |
| **3** | **Δ*G*_ele_, Δ*G*_vdW_** | 2 | 1 | 0.570 | 0.816 | 0.964 | 0.353 | 0.995 | 0.521 | 2 | -2 | 0.649 | 0.828 | 0.972 | 0.503 | 0.994 | 0.630 |
| **4** | **Δ*G*_GB_, Δ*G*_SA_** | 1.5 | 2 | 0.442 | 0.663 | 0.960 | 0.294 | 0.991 | 0.421 | 5 | -1 | 0.520 | 0.601 | 0.967 | 0.428 | 0.984 | 0.484 |
| **5** | **Δ*G*_ele_, Δ*G*_vdW_, Δ*G*_GB_, Δ*G*_SA_** | 3 | -0.5 | 0.634 | 0.723 | 0.970 | 0.468 | 0.990 | 0.563 | 2 | -2.5 | 0.714 | 0.844 | 0.974 | 0.537 | 0.994 | 0.659 |
| **Top 25 contributed residues** | | | | | | | | | | | | | | | | | |
| **6** | **Δ*G*_vdW_, Δ*G*_SA_** | 1.5 | 2 | 0.476 | 0.694 | 0.965 | 0.383 | 0.990 | 0.496 | 7 | -1.5 | 0.577 | 0.551 | 0.972 | 0.512 | 0.976 | 0.505 |
| **7** | **Δ*G*_ele_, Δ*G*_GB_** | 3 | -1 | 0.482 | 0.598 | 0.965 | 0.378 | 0.985 | 0.452 | 2.5 | -1.5 | 0.598 | 0.873 | 0.972 | 0.512 | 0.996 | 0.655 |
| **8** | **Δ*G*_ele_, Δ*G*_vdW_** | 2.5 | -1 | 0.551 | 0.695 | 0.969 | 0.453 | 0.989 | 0.541 | 2.5 | -2 | 0.684 | 0.753 | 0.974 | 0.547 | 0.990 | 0.625 |
| **9** | **Δ*G*_GB_, Δ*G*_SA_** | 7.5 | -1.5 | 0.444 | 0.479 | 0.968 | 0.453 | 0.972 | 0.436 | 2.5 | 0.5 | 0.590 | 0.731 | 0.970 | 0.473 | 0.990 | 0.569 |
| **10** | **Δ*G*_ele_, Δ*G*_vdW_, Δ*G*_GB_, Δ*G*_SA_** | 5 | -4 | 0.602 | 0.696 | 0.970 | 0.478 | 0.988 | 0.557 | 3 | -3.5 | 0.716 | 0.796 | 0.975 | 0.562 | 0.992 | 0.653 |
| **Top 30 contributed residues** | | | | | | | | | | | | | | | | | |
| **11** | **Δ*G*_vdW_, Δ*G*_SA_** | 1.5 | 2 | 0.488 | 0.697 | 0.965 | 0.378 | 0.991 | 0.494 | 7 | -1.5 | 0.577 | 0.553 | 0.972 | 0.517 | 0.976 | 0.509 |
| **12** | **Δ*G*_ele_, Δ*G*_GB_** | 1.5 | -0.5 | 0.499 | 0.697 | 0.961 | 0.309 | 0.992 | 0.445 | 2.5 | -2 | 0.595 | 0.884 | 0.971 | 0.493 | 0.996 | 0.647 |
| **13** | **Δ*G*_ele_, Δ*G*_vdW_** | 2 | -1.5 | 0.562 | 0.717 | 0.966 | 0.403 | 0.991 | 0.519 | 2.5 | -2 | 0.682 | 0.769 | 0.974 | 0.547 | 0.991 | 0.632 |
| **14** | **Δ*G*_GB_, Δ*G*_SA_** | 4.5 | -0.5 | 0.476 | 0.550 | 0.967 | 0.413 | 0.980 | 0.451 | 2 | 1 | 0.602 | 0.800 | 0.969 | 0.458 | 0.993 | 0.589 |
| **15** | **Δ*G*_ele_, Δ*G*_vdW_, Δ*G*_GB_, Δ*G*_SA_** | 8 | -5.5 | 0.598 | 0.663 | 0.974 | 0.547 | 0.984 | 0.582 | 2.5 | -3 | 0.727 | 0.812 | 0.975 | 0.557 | 0.993 | 0.658 |

**Table S5.** Detailed information of the 50 tested compounds in each group.

| **MIEC-SVM** | | | | **Autodock** | | | |
| --- | --- | --- | --- | --- | --- | --- | --- |
| **Specs ID*^a^*** | **Rank*^b^*** | **Averaged Enzymatic Inhibition @ 2ug/ml (%)** | **IC_50_** | **Specs ID** | **Rank** | **Averaged Enzymatic Inhibition @ 2ug/ml (%)** | **IC_50_** |
| AK-968/12265292 | 1 | -4.47 | - | AF-399/15128092 | 2 | -33.405 | - |
| AG-690/11765341 | 2 | 9.265 | - | AF-399/40960395 | 9 | -4 | - |
| AE-848/15116597 | 3 | 8.9 | - | AP-866/40876225 | 13 | 9.84 | - |
| AM-807/12426163 | 4 | 5.915 | - | AK-968/15362399 | 46 | 80.565 | 0.572 |
| AE-848/15116593 | 5 | -8.375 | - | AK-830/13217169 | 47 | 2.08 | - |
| AQ-432/43400100 | 6 | -6.36 | - | AE-848/15341192 | 48 | 13.145 | - |
| AQ-390/43364010 | 7 | 53.275 | 4.45 | AK-968/11200121 | 49 | -0.805 | - |
| AN-848/43256373 | 8 | -5 | - | AG-690/33068064 | 57 | 4.62 | - |
| AK-778/41314821 | 9 | -1.935 | - | AE-848/32007021 | 58 | 5.085 | - |
| AQ-390/42425809 | 10 | 83.015 | 0.513 | AF-399/15128132 | 63 | -9.645 | - |
| AH-487/37011044 | 11 | -17.16 | - | AG-690/12134081 | 67 | 10.575 | - |
| AF-399/40881300 | 12 | 13.735 | - | AG-690/11426045 | 78 | 50.43 | 0.832 |
| AF-399/14864072 | 13 | -20.25 | - | AQ-344/43100304 | 81 | -14.115 | - |
| AM-900/40673285 | 14 | 70.5 | 0.863 | AM-807/14959628 | 83 | -12.705 | - |
| AG-690/11836103 | 15 | -16.78 | - | AG-690/40750112 | 91 | 16.665 | -- |
| AQ-968/42007731 | 16 | -5.865 | - | AK-968/37077046 | 97 | 3.24 | - |
| AK-778/40897568 | 17 | 18.54 | - | AH-034/08463011 | 98 | 1.2 | - |
| AK-968/11780042 | 18 | -8.175 | - | AK-968/13025057 | 105 | 30.35 | - |
| AN-465/14952108 | 19 | 91.485 | 0.515 | AN-979/41713847 | 108 | 5.385 | - |
| AQ-390/42708910 | 20 | 75.485 | 0.359 | AK-918/15391011 | 109 | -13.24 | - |
| AE-848/15116611 | 21 | 10.08 | - | AG-690/40749597 | 115 | 2.49 | - |
| AQ-390/40910467 | 23 | 50.76 | 6.052 | AN-919/13953019 | 118 | 65.775 | 1.535 |
| AN-648/40740338 | 24 | 23.375 | - | AF-399/42017504 | 119 | -5.97 | - |
| AG-205/33688061 | 28 | 14.9 | - | AB-131/42301451 | 120 | 28.575 | - |
| AO-081/15572353 | 31 | -3.26 | - | AG-690/36541063 | 133 | 11.335 | - |
| AO-476/43362608 | 32 | 9.055 | - | AG-690/12869680 | 135 | -1.245 | - |
| AG-664/32341038 | 33 | 6.53 | - | AN-465/40934866 | 139 | 2.22 | - |
| AN-512/12594125 | 34 | 10.48 | - | AK-968/12097078 | 141 | -2.025 | - |
| AL-182/40744490 | 35 | -4.165 | - | AG-690/40749514 | 157 | -4.41 | - |
| AO-080/13867269 | 36 | 64.86 | 1.531 | AN-465/13859038 | 159 | 40.815 | - |
| AO-080/43378230 | 38 | -9.72 | - | AF-399/15128126 | 160 | 32.1 | - |
| AS-871/43476359 | 39 | 48.88 | 11.529 | AF-399/15128108 | 162 | -21.105 | - |
| AS-871/42941985 | 40 | -10.47 | - | AK-968/11842315 | 164 | 10.91 | - |
| AO-081/15045283 | 41 | 37.24 | 18.232 | AK-918/13399094 | 173 | 8.005 | - |
| AN-829/42007480 | 42 | 2.755 | - | AG-690/12869023 | 178 | 12.235 | - |
| AE-641/40974923 | 43 | 13.995 | - | AH-541/12737056 | 182 | 12.4 | - |
| AO-081/15386972 | 45 | -1.4 | - | AI-204/31728031 | 184 | 3.565 | - |
| AN-465/14954307 | 46 | 46.45 | - | AG-690/40097114 | 186 | 1.725 | - |
| AN-652/43161615 | 47 | 17.89 | - | AA-504/07472022 | 188 | 40.455 | - |
| AS-871/41399397 | 48 | -8.995 | - | AG-690/09396003 | 194 | 3.685 | - |
| AM-879/41780072 | 49 | 12.385 | - | AK-968/15360205 | 198 | -17.96 | - |
| AF-399/15335536 | 51 | 5.725 | - | AN-655/13300050 | 206 | 23.865 | - |
| AK-968/37055166 | 52 | -8.735 | - | AJ-292/42284747 | 212 | 1.31 | - |
| AG-205/37086057 | 53 | 40.815 | - | AG-205/36717014 | 218 | 19.28 | - |
| AF-399/14388040 | 54 | -20.41 | - | AQ-750/42209760 | 219 | 13.425 | - |
| AG-205/36915079 | 56 | 18.19 | - | AE-848/12917024 | 220 | 13.565 | - |
| AG-690/08629011 | 57 | 26.47 | - | AN-789/13819006 | 223 | 1.595 | - |
| AN-655/15262058 | 58 | 11.82 | - | AG-690/40097127 | 230 | 11.435 | - |
| AG-690/40751357 | 59 | 8.965 | - | AP-964/41446891 | 233 | 13.75 | - |
| AH-487/40936079 | 60 | 0.44 | - | AG-690/37079179 | 237 | 15.845 |  |

*^a^*According to the purity statements, the purity of all substances purchased from Specs is higher than 95%.

*^b^*Ranks derived from MIE-SVM inhibitor-probability and docking score.
